# Supplementary material for: A whole-animal phenotypic drug screen identifies suppressors of atherogenic lipoproteins
Source: eLife. 2026 Aug 27;14:RP105314. doi: 10.7554/eLife.105314 (PMC13521566; doi:10.7554/eLife.105314)
Supplement: Supplementary file 2 [file elife-105314-code1.zip › JHDL_validation_result/Medroxyprogesterone_UK20210419_01.pdf]

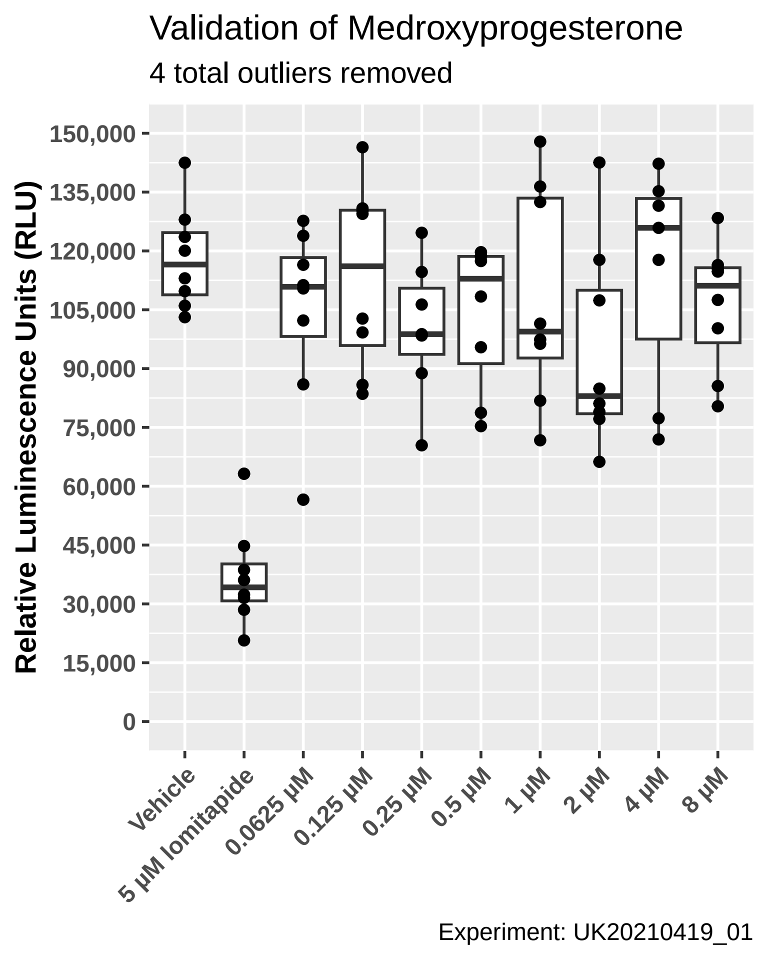
Validation of Medroxyprogesterone
4 total outliers removed
Relative Luminescence Units (RLU)
150,000
135,000
120,000
105,000
90,000
75,000
60,000
45,000
30,000
15,000
0
Vehicle
5 µM lomitapide
0.0625 µM
0.125 µM
0.25 µM
0.5 µM
1 µM
2 µM
4 µM
8 µM
Experiment: UK20210419_01
